# Preliminary study of transoral robotic surgery for pharyngeal cancer in Japan

**DOI:** 10.1007/s11701-015-0547-7

**Published:** 2015-12-08

**Authors:** Kazunori Fujiwara, Takahiro Fukuhara, Hiroya Kitano, Taihei Fujii, Satoshi Koyama, Aigo Yamasaki, Hideyuki Kataoka, Hiromi Takeuchi

**Affiliations:** Department of Otolaryngology, Head and Neck Surgery, Faculty of Medicine, Tottori University, 36-1, Nishimachi, Yonago, 683-8504 Japan

**Keywords:** Transoral robotic surgery (TORS), Pharyngeal cancer, Head and neck cancer, Single-institute clinical trail

## Abstract

Transoral robotic surgery (TORS) with the da Vinci Surgical System has been used for the removal of pharyngeal and laryngeal cancers with the objective to improve functional and aesthetic outcomes without worsening survival. While TORS has been approved in many countries, Japan’s FDA has not yet done so. Our hospital started using TORS with the approval of the Ethical Review Board and the Minimum Invasive Surgical Center Committee at Tottori University. No surgical outcomes of TORS for Japanese patients with head and neck cancer have been reported in Japan. This paper deals with the outcomes and feasibility of TORS for Japanese patients with pharyngeal cancer at our institution. TORS was performed for 10 patients with T1, T2, T3 oropharyngeal and hypopharyngeal squamous cell carcinoma between 2013 and 2014. This is a single-institutional study. TORS could be completed for all cases, except one patient that was not candidate, and no intraoperative conversion to an open surgical procedure was required. Five patients underwent neck dissection, two of them concurrent and three staged. Of all patients, positive surgical margins were detected in two. The average blood loss including neck dissection was 21.5 ± 33.4 ml, the operation time was 183 ± 36 min and the console time was 103 ± 22 min. No tracheostomy had been performed either pre- or postoperatively, and there was no difference between preoperative and postoperative swallowing functions. In this single-institutional preliminary study, we demonstrated that TORS is a feasible and safe treatment. A clinical multi-institutional study of TORS for laryngopharyngeal cancer has been approved as an advanced medical system study and is under way. In the near future, it is expected that the efficacy and safety of TORS for laryngopharyngeal cancer will be confirmed as the result of this multiple-institutional clinical study in Japan.

## Introduction

Head and neck squamous cell carcinoma (HNSCC) is the sixth most prevalent neoplasm worldwide and 500,000 patients are diagnosed with HNSCC annually. Surgery, radiotherapy or chemotherapy, or a combination of these treatments, depending on location and stage, is used to treat HNSCC. Recently, a trend has been observed to use radiotherapy and concurrent chemotherapy as a primary modality in case of early stage oropharyngeal cancer [1, 2]. Meta-analysis data have demonstrated not only improved survival with altered fractionated regimens and/or the addition of chemotherapy, but also a significant increase in treatment-related toxicities, particularly acute mucositis xerostomia and long-term swallowing dysfunction [3–5]. The rate of gastrostomy tube dependence for patients treated with chemoradiotherapy has been reported as typically between 9 and 39 % in patients treated with chemoradiotherapy [6].

Minimally invasive surgery (MIS) techniques for HNSCC continue to be frequently reported in the head and neck literature, driven by the desire to offer a less morbid alternative to chemoradiation. These techniques have included transoral laser microsurgery (TLM) and more recently transoral robotic surgery (TORS). TORS was first introduced by Weinstein et al. [7] in 2005 with their case report of supraglottic laryngectomy performed in a canine model.

Transoral robotic surgery (TORS) with the da Vinci Surgical System has been used for the removal of pharyngeal and laryngeal cancers with the objective to improve functional and aesthetic outcomes without worsening survival [6, 8, 9]. On the basis of favorable reports concerning transoral laser microsurgery (TLM), the benefits of the transoral approach to the pharyngo-laryngeal lumen are well known [10, 11]. TORS allows for a clearer and wider view of the surgical field and better 3D visualization of structures than TLM, making access to the tumor possible via an approach smaller than the external one. Another advantage of TORS is the use of miniaturized tools, which allows for mimicking standard surgical instruments and arm movements but with tremor filtration. Additionally, robotic surgery does not require line-of-site as in the case of CO2 laser microlaryngeal surgery. Therefore, TORS has been developed with feasibility studies confirming the maintenance of swallowing function, effective local control and usability of this procedure for head and neck cancer [12–15].

While TORS has been approved in many countries, Japan’s FDA has not yet done so. Our hospital started using TORS with the approval of the Ethical Review Board and the Minimum Invasive Surgical Center Committee at Tottori University. No surgical outcomes of TORS for Japanese patients with head and neck cancer have been reported in Japan. This paper deals with the outcomes and feasibility of TORS for Japanese patients with pharyngeal cancer at our institution.

## Methods

Between January 2013 and December 2014, 11 patients with various stages of HNSCC participated in a clinical trial of transoral robotic surgery for pharyngeal cancer. The Tottori University institutional review board’s (IRB) approval was obtained to use the da Vinci Surgical System (Intuitive Surgical Inc., Sunnyvale, CA) for resection of head and neck cancer. 2723 informed consent was received from all patients, and those who were included in this study had been prospectively enrolled based on the protocol approved by the Tottori University IRB. Preoperatively, all patients underwent endoscopic examination, a contrast-computed tomography of their throat, neck and chest, and ultrasonography of their neck as well as a PET–CT whole body scan except for patients with diabetes mellitus. We measured the mouth opening and performed cephalometry to determine whether a patient had trismus or brachygnathia.

For the purpose of this study, we selected patients with the following characteristics: (1) aged 20 years or older, (2) diagnosis of oropharyngeal or hypopharyngeal squamous cell carcinoma, classified preoperatively as T1 or T2 according to the UICC classification 2010 [16], (3) performance status 0 or 1, (4) approval by the Tottori University Minimum Invasive Surgical Center Committee. Exclusion criteria included patients with poor mouth opening that would preclude adequate exposure of the affected area.

Only one surgeon approved by the Tottori University Minimum Invasive Surgical Center Committee performed the TORS console procedure for this study. The surgeon and an assistant underwent basic training to obtain certification as console surgeon for the da Vinci surgical system as well as advanced training using both animal and cadaveric dissection. The console surgeon and the assistant (both head and neck surgeons), an anesthesiologist, a clinical engineering technologist, and operating nurses cooperated for preoperative simulation of the robotic setting and surgical process.

TORS procedures were performed under general anesthesia as described elsewhere in detail [15]. Patients underwent the surgery at the Minimum Invasive Surgical Center, Tottori University. If neck ultrasonography showed the presence of neck metastasis, neck dissection was performed as a concurrent and staged procedure depending on the primary tumor site. The patient was operated on in the supine position. Patients with oropharyngeal cancer were intubated nasopharyngeally, those with hypopharyngeal cancer were intubated orotracheally. A FK-WO TORS laryngo-pharyngoscope retractor (Olympus, Tokyo, Japan) was positioned so as to expose the primary tumor and to provide sufficient working space. The retractor was then suspended with a holder and the da Vinci cart was positioned on the right side and at a 45° angle to the operating table. The teeth were covered with a mouthpiece made to order at our dental clinic from polyethylene terephthalate glycol (Erkodur). A 3D endoscope was inserted through the oral cavity and two articulated robotic instruments were inserted on each side of the endoscope. A 0° 3D endoscope was selected for the soft palate and lateral wall of the oropharynx, and a 30° 3D endoscope for the tongue base and hypopharynx. As for instruments, a 5 mm monopolar spatula was used on the affected side, and Maryland forceps were used on the intact side. The instrument might be switched with the other side, depending on the situation. The tumor margin was determined with the aid of a narrow band image, and the resection was performed en bloc. To confirm adequate resection, frozen sections were examined followed by additional resection when necessary.

The endpoint included positive rate of the surgical margin, operation time, surgical feasibility, postoperative swallowing function, and gastrointestinal tube rate. Feasibility was measured in terms of the capability for TORS procedures to be performed without the need for conversion to a non-robotic approach. The adequacy of surgical margins was determined in terms of the presence of any carcinoma or carcinoma in situ at the conclusion of the final pathological examination. Discontinuance criteria were: more than 500 ml blood loss, more than 3 h console time, and sudden worsening of general condition.

If multiple neck lymph node metastases were found, postoperative radiotherapy was performed, and if extra-nodal spread of the neck lymph node or a positive margin of the primary tumor was detected, postoperative concurrent chemoradiotherapy was used.

## Results

### Patients and tumor characteristics

Between January 2013 and March 2015, 11 patients with various stages of HNSCC participated in a clinical trial of transoral robotic surgery for pharyngeal cancer. One of the 11 recruited patients was found not to be a candidate for TORS because the affected area could not be adequately exposed. The patient’s mouth opening was 26 mm and cephalometry showed brachygnathia. We also checked the robotic setting using the FKWO retractor under general anesthesia and found it difficult to secure the surgical field. Eventually, chemoradiotherapy was selected for this patient. In all cases except one patient who did not qualify as a candidate, the TORS procedure could be completed and without the need for intraoperative conversion to an open surgical procedure.

The average age of the subjects was 68 years, 9 were male and 1 was female. Performance status (PS) was 2 for 1 patient and 0–1 for the others. Most patients had squamous cell carcinoma, and one patient with hypopharyngeal cancer had an undifferentiated carcinoma. Two patients had undergone a previous resection without radiotherapy for oral and oropharyngeal carcinoma, one had undergone a previous resection (total laryngopharyngectomy) and chemoradiotherapy for oropharyngeal carcinoma one had undergone a previous resection (total laryngopharyngectomy) without adjuvant therapy and the remaining patients had not undergone any previous treatment. The anatomic site locations of the cancers are shown in Table 1. T classification was T1 for four patients, T2 for five patients, and T3 for one patient. There were no T is patients. Five patients underwent neck dissection, patients concurrent for two and staged for three.

**Table 1:** Primary characteristics of 10 TORS cases

Case	Age	Sex	Disease	Lesion	TNM	Past history
1	70	M	Oropharynx	Lateral	T2N0M0 (double)	Hypopharyngeal cancer, cerebral infarction, DM, HT
2	60	F	Oropharynx	Lateral	T1N0M0 (recurrent)	Oral cancer, HT
3	87	M	Oropharynx	Tongue base	T1N2bM0 (primary)	DM, HT
4	60	M	Hypopharynx	Post	T2N2cM0 (primary)	HT
5	71	M	Oropharynx	Lateral	T1N0M0 (double)	Oral cancer
6	66	M	Hypopharynx	Piriform	T1N1M0 (primary)	Macrogloblin
7	63	M	Hypopharynx	Piriform	T2N0M0 (primary)	HT
8	68	M	Oropharynx	Lateral	T3N2bM0 (primary)	DM
9	63	M	Oropharynx	Lateral	T2N1M0 (primary)	Bladder cancer
10	56	M	Oropharynx	Soft palate	T2N0M0 (recurrent)	Alcohol dependence, oropharyngeal cancer

*DM* diabetes, *HT* hypertension

### Perioperative outcome

Positive surgical margins were detected in only two patients. One patient had recurrent oropharyngeal cancer, and the surgical margin was positive and vertical. The other patient had hypopharyngeal cancer and the surgical margin was positive and horizontal (Table 2).

**Table 2:** Surgical outcomes

Case	Disease	Bleeding	Operation time	Console time	Margin	Neck dissection
1	Oropharynx	0	262	150	Negative	–
2	Oropharynx	15	216	100	Positive (vertical)	–
3	Oropharynx	25	165	110	Negative	Rt RND concurrent
4	Hypopharynx	35	149	68	Negative	Bil MND staged
5	Oropharynx	0	186	90	Negative	–
6	Hypopharynx	110	138	93	Negative	Rt SND concurrent
7	Hypopharynx	20	201	120	Positive (horizontal)	–
8	Oropharynx	0	181	108	Negative	Rt SND staged
9	Oropharynx	0	161	100	Negative	Lt MND staged
10	Oropharynx	10	177	91	Negative	–
Average		21.5	183.6	103		

The average blood loss was 21.5 ± 33.4 ml, the average operation time was 183.6 ± 36.0 min, and the average console time was 103 ± 21.6 min

**Fig. 1:**
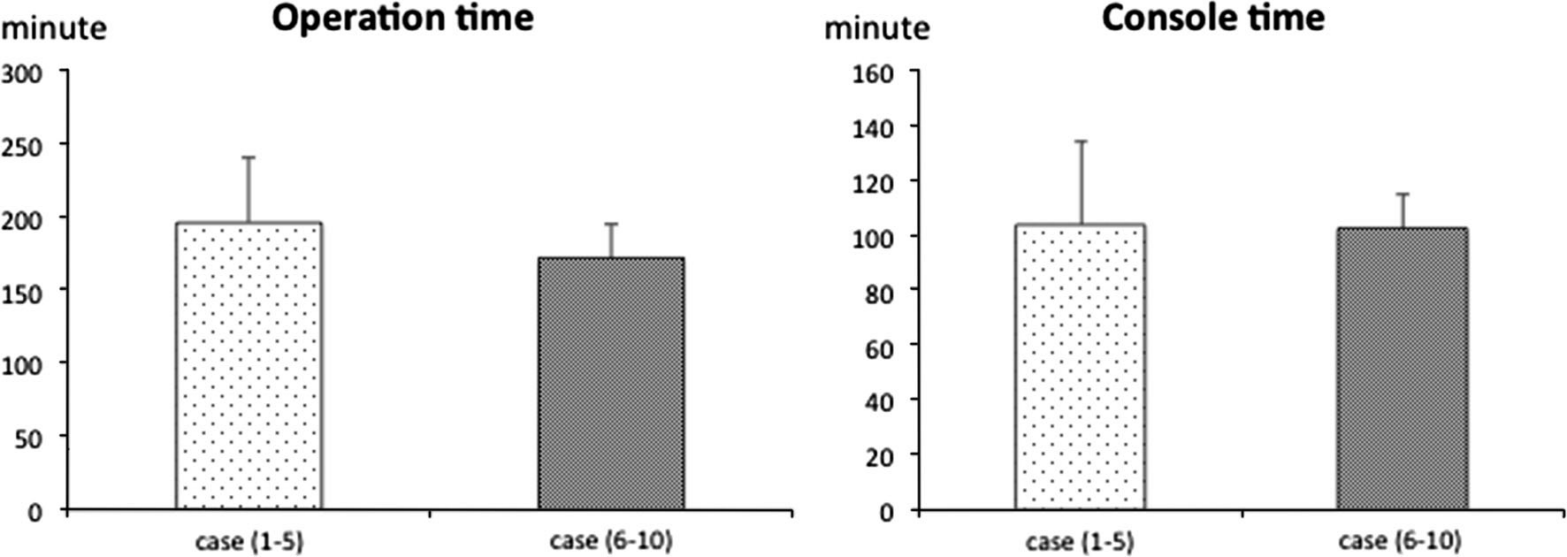
Operation time and console time. There is no difference between earlier and later cases in either operation or console time

**Fig. 2:**
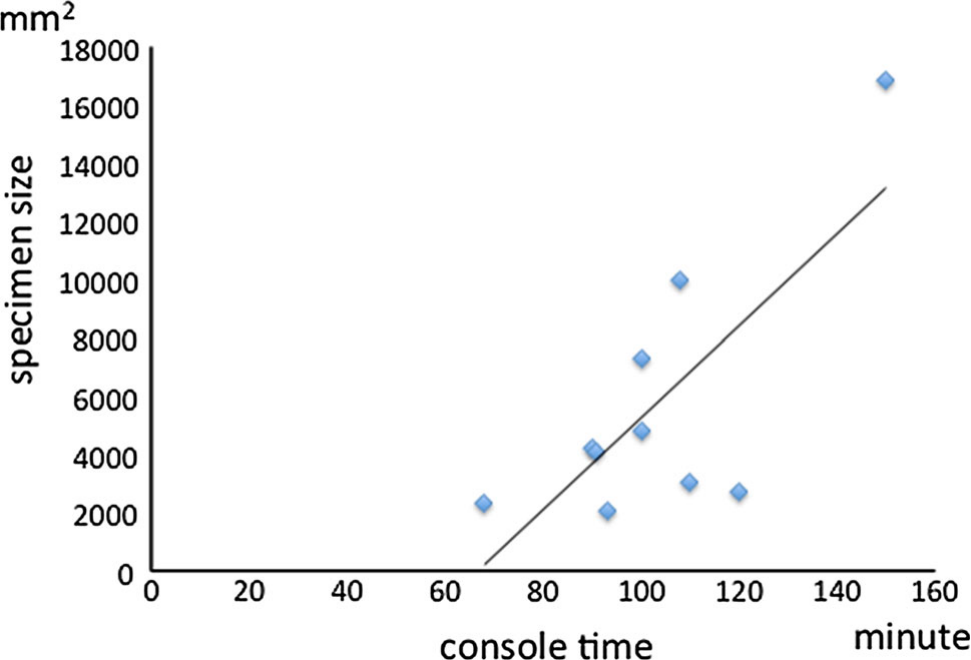
Correlation between specimen size and console time. Console time showed a significant correlation with specimen size (correlation coefficient: 0.75)

There were no intraoperative complications for any of the cases. Blood loss for all patients undergoing TORS including neck dissection was 21.5 ± 33.4 ml and none of them needed blood transfusion (Table 2). The average operation time for the TORS procedure including robotic setup without neck dissection was 183 ± 36 min. Robotic setup time included time to achieve adequate exposure, robotic system docking, and to determine the tumor margin using narrow-band imaging. The average console time was 103 ± 22 min and was spent during the robotic part of the procedure (Table 2). Console time included non-operative time waiting for frozen section results. There were no differences in either operation time or console time between the first five cases and the last five. Even as experience with TORS increased, surgical time and console time did not decrease (Fig. 1). Console time showed a significant correlation with specimen size (correlation coefficient: 0.75) (Fig. 2).

No tracheostomy was performed either perioperatively or postoperatively. The average length of hospital stay was 8 days (Table 3).

**Table 3:** Postoperative outcomes

Case	Hospital stay	Time to oral intake	Postoperative complication	Tracheostomy
1	7	3	–	Stoma
2	7	1	–	–
3	7	2	–	–
4	7	21	–	–
5	7	PEG	–	–
6	7	1	–	–
7	14	6	–	–
8	9	3	–	–
9	7	3	–	–
10	8	4	–	Stoma

Case 5 had no oral intake before TORS

*PEG* percutaneous endoscopic gastroscopy

### Adverse events

No intraoperative or perioperative fatalities occurred, nor was any catastrophic hemorrhage or emergent airway compromise observed. None of the patients experienced serious adverse events that either required hospitalization or intervention or were considered life threatening. Nine patients showed no postoperative swallowing function abnormalities with a functional outcome swallowing scale (FOSS) of grade 0–1, and one patient had preoperative inadequate oral intake resulting from oral cancer resection (FOSS grade 3). No patient newly required gastrointestinal tube postoperatively. There was thus no change between preoperative and postoperative swallowing function. All patients were fed postoperatively via a nasogastric feeding tube, which was removed after a mean of 4.8 days (range 1–21 days) (Table 3).

In short, there were no serious adverse events judged to be directly attributable to the robotic device, nor was there any instrument malfunction.

### Postoperative therapy

Postoperative radiation was administered to two patients, one with oropharyngeal cancer with a positive vertical margin (case No. 2) and one with hypopharyngeal cancer (case No. 8) with multiple lymph node metastases. Postoperative chemoradiation was administered in one case of hypopharyngeal cancer (case No. 7) with a positive horizontal margin. The radiation dose for all cases was 66 Gy and all radiotherapy and chemoradiotherapy courses were completed. However, the case treated with chemoradiotherapy showed temporary hyponatremia.

Local recurrence in the tongue base and oral floor was observed in the oropharyngeal cancer case with a positive vertical margin and additional resection. This patient underwent extensive oral oropharyngeal cancer resection and complete neck dissection after TORS.

Two recurrences in the contralateral neck lymph node were detected, one in a patient with hypopharyngeal cancer (case No. 6) and the other in a patient with oropharyngeal cancer (case No. 8). These patients underwent selective neck dissection. There were no instances of ipsilateral neck lymph node recurrence.

## Discussion

TORS with the da Vinci Surgical System has been used for the removal of pharyngeal and laryngeal cancers with the objective to improve functional and aesthetic outcomes without worsening survival [6, 8, 9]. TORS has been approved in many countries, and its safety and efficacy for laryngopharyngeal tumors has been reported. However, at present time, the Japanese Health, Labor and Welfare Ministry have not yet granted approval for the use of the da Vinci Surgical System for head and neck tumors. Our institution, therefore, started a clinical trial of TORS in 2013 with the approval of the Tottori University Review Board and the Tottori University Minimum Invasive Surgical Center Board for evaluation of the safety and efficacy of TORS for Japanese head and neck cancer patients. This report thus concerns a preliminary single-institution study in Japan and deals with the feasibility, safety, surgical margin, postoperative swallowing function, and adverse event of TORS for head and neck cancer. This is the first report on a clinical study of TORS in Japan.

In all cases, except for one patient who did not qualify as a candidate because the affected area could not be adequately exposed, the TORS procedure could be completed and no intraoperative conversion to an open surgical procedure was required despite strict discontinuance criteria. The body structure and shape of the skull of Japanese people tend to differ from those of other racial groups, especially Caucasian, so that difficulties were expected with using the TORS procedure for Japanese patients. However, our study was performed safely and completely. A multi-institutional study reported that 1.1 % of patients who underwent the TORS procedure required conversion to open surgery [17]. TORS thus involves a low rate of conversion to open surgery and a high completion rate in comparison with other transoral surgerical procedures, especially transoral laser surgery. All the patients in our study were checked for trismus and brachygnathia to determine if they were suitable for TORS, and several institutions performed preoperative assessment for suitability for robotic surgery under general anesthesia [18]. To maintain a high completion rate, preoperative assessment of potential problems associated with tumor exposure might be required. We hope to develop an easy and objective preoperative assessment for suitability of TORS, partly because the cost of robotic surgery is comparatively high.

Surgical time for our study was 183 min ± 36 min and console time 103 ± 22 min. Richmon et al. reported that their surgical time averaged 242 ± 84 min and console time 71 ± 54 min, while a multi-center study reported that the average time for TORS procedure, including robotic setup, was 167.5 ± 54.5 min not including concurrent neck dissection. Console time for our study included nonoperative time waiting for frozen section results. In addition, we detected early superficial pharyngeal cancers using narrow band imaging. It was reported that the combination of TORS and narrow band imaging for determining the extent of resection was useful [19]. Although this procedure requires additional time, we consider it necessary for preventing positive margins.

In the present study, there was no significant difference in operation time and console time between the earlier and later cases and correlation of console time with specimen size was significant. Another study reported that there were no significant differences in either of the two times between the initial and subsequent 10 cases or the first 15 and last 5 cases [20]. On the other hand, White et al. [21] reported that the operating time decreased as experience with TORS increased. However, the da Vinci surgical system provides a simulation system for training of operators. Also, the surgeon at our hospital had not only obtained certification for the da Vinci surgical system but also taken an advanced training course including cadaver dissection. The lack of significant shortening and rapid achievement of operation technique demonstrated that adequate training and preparation before initiation of TORS resulted in optimal efficiency.

Average blood loss for all patients undergoing TORS was 21.5 ml ± 33.4 ml and no surgical complications occurred. A multi-center study reported that blood loss was 82.8 ± 130 ml and 16 % patients experienced serious adverse events, such as pneumonia, dysphagia, and laryngeal edema, that required hospitalization or intervention (grade 3) or that were considered life threatening (grade 4). Also, 9 % of the patients experienced postoperative bleeding [17]. Our data demonstrate that TORS provided adequate safety and also suggest that an institutional supervisory and approval system may contribute to even safer surgery.

Two patients in this study showed a positive surgical margin, and just Published studies related to TORS reported that rates of inadequate or positive surgical margins mentioned in pathology reports were 0–33 % with local control rates of 91–100 % [9, 22–30]. However, strict criteria for margins have not been established yet. Since our study involved only a small number of cases, it is difficult to evaluate the results accurately and reliably, but that appear to be at least comparable to other data reported previously. To further improve surgical outcomes, we will continue to use the narrow band image and frozen section examination for TORS.

Kyoto University, Tottori University, and Tokyo Medical University applied to the Japan Health, Labor and Welfare Ministry to obtain approval for use of the da Vinci Surgical System for head and neck cancer for a clinical multi-institutional study of transoral robotic surgery for laryngopharyngeal cancer. Approval was granted in 2015, and the study was started as an advanced medical system study. In the near future, it is expected that the efficacy and safety of TORS for laryngopharyngeal cancer will be confirmed as the result of this first multiple-institutional study in Japan.
